# Monitoring Walker Assistive Devices: A Novel Approach Based on Load Cells and Optical Distance Measurements †

**DOI:** 10.3390/s18020540

**Published:** 2018-02-10

**Authors:** Vítor Viegas, J. M. Dias Pereira, Octavian Postolache, Pedro Silva Girão

**Affiliations:** 1 Instituto de Telecomunicações, 1049-001 Lisboa, Portugal; opostolache@lx.it.pt (O.P.); psgirao@tecnico.ulisboa.pt (P.S.G.); 2 ESTSetúbal, Instituto Politécnico de Setúbal, 2910-761 Setúbal, Portugal; dias.pereira@estsetubal.ips.pt; 3 ISCTE—Instituto Universitário de Lisboa, 1600-077 Lisboa, Portugal; 4 Instituto Superior Técnico, Universidade de Lisboa, 1049-001 Lisboa, Portugal

**Keywords:** force measurement, distance measurement, walker assistive device, gait analysis, usage monitoring

## Abstract

This paper presents a measurement system intended to monitor the usage of walker assistive devices. The goal is to guide the user in the correct use of the device in order to prevent risky situations and maximize comfort. Two risk indicators are defined: one related to force unbalance and the other related to motor incoordination. Force unbalance is measured by load cells attached to the walker legs, while motor incoordination is estimated by synchronizing force measurements with distance data provided by an optical sensor. The measurement system is equipped with a Bluetooth link that enables local supervision on a computer or tablet. Calibration and experimental results are included in the paper.

## 1. Introduction

It is estimated that by the year 2025, in the United States and Canada, 25% of the population will be over 65 years old [1,2,3]. Moreover, it is expected that in the European Union the life expectance for the year 2060, for women and men, will be around 89 and 84.5 years, respectively [4]. Thus, topics related with the mobility of elderly people have an increased importance, particularly in which concerns the proper usage of mobility aiding devices [5,6]. The careful and conscientious use of these devices can avoid harmful injuries [7,8,9], namely the ones caused by elderly people falls. Besides the improvements related with elderly people, in terms of quality of life and extension of the time they can live autonomously at home, mobility adding devices are also very important for patients during rehabilitation processes.

Several solutions were developed to address these problems [10,11,12,13,14] but many of them are too complicated, too expensive or too impractical for day-to-day applications. Many solutions make use of accelerometers and Inertial Motion Units (IMU) to extract kinematic parameters related with human gait [15,16,17]. However, these solutions require complex algorithms to improve measurement accuracy, in particular to mitigate the problems related with time integration of signals that contain persistent DC offsets (as it happens with low-cost accelerometers). 

Solutions based on RAdio Detection and Ranging (RADAR) measurements are also referred to in the literature [18,19] and they are successful regarding the identification of gait patterns but not so efficient to obtain accurate measurements of walking distance. 

Usage of ultrasounds can also be a solution to obtain distance measurements [20]. However, ultrasound measurements are not directional and are affected by external factors such as environmental temperature and humidity.

Several works [21,22] also refer the usage of image processing techniques to monitor gait. The main advantages of these methods are related with their non-invasiveness but there are strong limitations related with camera positioning and it is not possible, at all, to acquire data concerning force intensities.

Concerning force measurements, used to evaluate walking balance conditions, the authors already reported a measurement system that uses Force Sensing Resistors (FSR) [23]. The results were acceptable but the main problems identified were related with lack of repeatability, poor robustness and sensors’ detrition. The positioning of the FSRs at the bottom of walker legs was critical to assure a uniform pressure distribution over the sensitive area, thus affecting the repeatability of the measurements over time.

To overcome some of these limitations and to contribute to further advances in this research area, the authors propose a low-cost, easy-adaptable instrumented walker that detects risk situations motivated by unbalance and motor incoordination. This new proposal makes use of load cells to measure the force applied on the walker legs and Light Detection and Ranging (LIDAR) to measure distance. The acquired data is transferred through a Bluetooth link to a personal computer where it is processed and presented to a physiotherapist.

A prototype based on a conventional walker with a four legs ground contact configuration was implemented and used for testing purposes. The measurement methods and technical solutions presented here can easily be applied to other mobility aiding devices, particularly walkers with different ground contact configurations such as wheeled walkers and rollators.

The paper is organized as follows: Section 2 defines metrics to assess risk; Section 3 describes the implemented measurement system; Section 4 presents experimental results; and Section 5 draws conclusions.

## 2. Usage Metrics

Two risk indicators, one related with force unbalance (*I*_1_) and the other related with motor incoordination (*I*_2_), are defined to monitor walker usage and detect potential dangerous situations. 

### 2.1. Force Balance

**Figure 1:**
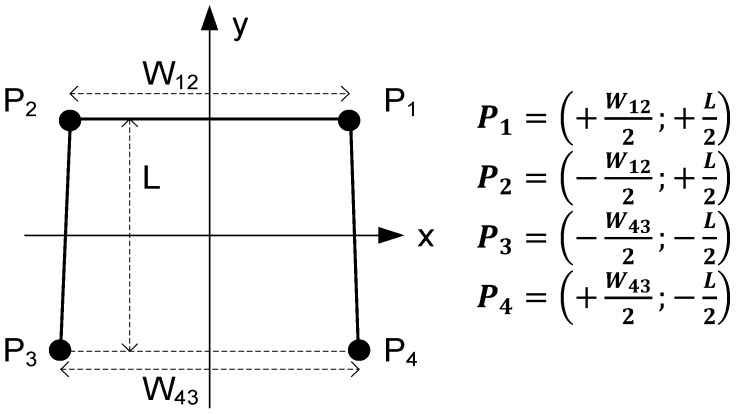
Coordinate system chosen for the measurement system. The black circles represent the walker legs (numbered as the quadrants in the Cartesian plane), *P*_k_ represents the application point of force *F*_k_ and the *y* axis indicates the direction of movement.

The first risk indicator (*I*_1_) has to do with the (un)balance of forces applied on the walker legs. Considerer the coordinate system illustrated in Figure 1 where the walker legs are numbered from 1 to 4 (as quadrants) and the *y*-axis points to the forward direction. According to this arrangement, the Centre of Forces (*COF*) is given by [24]:

(1) $$COF_{x}=\frac{W_{12}\left(F_{1}-F_{2}\right)+W_{43}\left(F_{4}-F_{3}\right)}{2\left(F_{1}+F_{2}+F_{3}+F_{4}\right)}$$

(2) $$COF_{y}=\frac{L\left(F_{1}-F_{4}\right)+L\left(F_{2}-F_{3}\right)}{2\left(F_{1}+F_{2}+F_{3}+F_{4}\right)}$$

where *F_k_* represents the magnitude of the force applied to each leg, *W*_12_ and *W*_43_ represent the distances between the front and rear legs respectively—which corresponds roughly to the mean walker width (*W*), *L* represents the distance between front and rear legs, which corresponds to the walker length.

The unbalance indicator is defined as the deviation of the *COF* in relation to the geometrical centre of the walker polygon:

(3) $$I_{1}\left(\%\right)=100\times \frac{\sqrt{{\left(COF_{x}\right)}^{2}+{\left(COF_{y}\right)}^{2}}}{\sqrt{{\left(W/2\right)}^{2}+{\left(L/2\right)}^{2}}}\times \alpha$$

with α being given by:

(4) $$\alpha =\frac{F_{1}+F_{2}+F_{3}+F_{4}}{F_{U}}$$

where the numerator represents the total force applied on the walker legs and *F_U_* represents the user weight. Alpha (*α*) is a weighting factor that takes values between 0 (when the walker is resting) and 1 (when the walker is charged with all the weight of the user).

### 2.2. Motor Coordination

**Figure 2:**
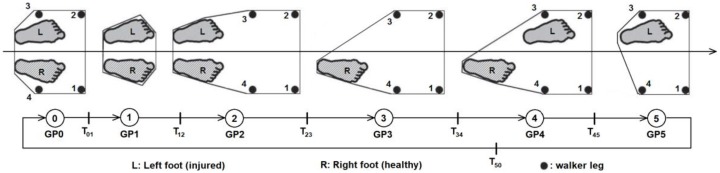
Gait Phases (GP) during a walker step (the polygon delimits the support area). GP0: the walker is resting on the floor; GP1: the walker is flying; GP2: the walker is on the floor waiting for the injured foot to move forward; GP3: the injured foot is moving forward; GP4: waiting for the healthy foot to move forward; GP5: the healthy foot is moving forward.

The second risk indicator (*I*_2_) has to do with the (in)coordination between walker movements and user gait. Considerer the state machine illustrated in Figure 2, first proposed by Winter D.A. [25,26], where the states represent the Gait Phases (GP) and the continuous arrows represent normal transitions between states.

The transitions are validated by sensor measurements as follows:

- T_01_ is enabled when *F*_1_ + *F*_2_ + *F*_3_ + *F*_4_ < WEIGHT_TH (threshold of minimum weight). This transition occurs when the total force measured by the load cells falls below the walker weight; in other words, when the walker is lifted in the air.
- T_12_ is enabled if *F*_1_ + *F*_2_ + *F*_3_ + *F*_4_ > WEIGHT_TH AND *d* > TRAVEL_TH (threshold of minimum distance travelled forward). This transaction occurs when the walker touches the floor after traveling a distance greater than the minimum.
- T_23_ is enabled if *COF_x_* > RIGHT_TH or *COF_x_* < LEFT_TH depending on which foot is injured (left or right, respectively). This transaction occurs when the user steps forward with the injured foot and applies maximum force on the opposite side. The injured foot shall always be the first one to move. The type of disability must be defined in advance because it determines the threshold value.
- T_34_ is enabled if *COF_x_* < RIGHT_TH or *COF_x_* > LEFT_TH depending on which foot is injured (left or right, respectively). This transaction occurs when the user alleviates the force previously applied to the walker; in other words, when *COF_x_* returns to zero.
- T_45_ is enabled if *COF_x_* < LEFT_TH or *COF_x_* > RIGHT_TH depending on which foot is injured (left or right, respectively). This transaction occurs when the user steps forward with the healthy foot and applies maximum force on the opposite side. The healthy foot shall always be the last one to move.
- T_50_ is enabled if *COF_x_* > LEFT_TH or *COF_x_* < RIGHT_TH depending on which foot is injured (left or right, respectively). This transaction occurs when the user alleviates the force previously applied to the walker; in other words, when *COF_x_* returns to zero.

If the machine passes through all the states successfully then the step is marked as “good”; otherwise, the step is marked as “bad” and the counter *B* is incremented. The incoordination indicator is then computed as:

(5) $$I_{2}\left(\%\right)=100\times \frac{B}{N}$$

where *N* is a moving window covering the last steps (defaults to 10).

## 3. Measurement System

The measurement system includes four load cells to sense the force applied on the walker legs, a LIDAR to measure the travelled distance, a data acquisition board with Bluetooth link and software to process and present data. The apparatus was installed on a pick-up standard walker [27] with the following characteristics: width between front legs (*W*_12_) equal to 52 cm, width between rear legs (*W*_43_) equal to 53 cm, length between front and rear legs (*L*) equal to 45 cm, adjustable height between 78 cm and 90 cm in increments of 2.5 cm.

### 3.1. Force Sensors

Force measurements were initially done by FSRs as described in [23]. Unfortunately, the installation of these sensors was very tricky making it very difficult to guarantee that each sensor had the same mechanical coupling to the process and, consequently, the same sensitivity. To overcome this problem, FSRs were replaced by load cells. 

**Figure 3:**
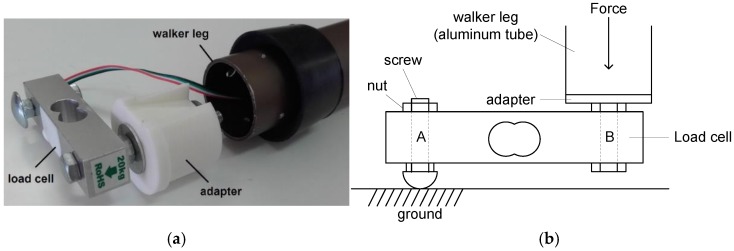
Load cell attached to the walker leg: (**a**) Photo; (**b**) Side view.

Each load cell was attached to the extremity of a walker leg using a dedicated plastic adapter grown on a low-cost 3D printer (see Figure 3). The cell contains four strain gauges fixed to a small body of aluminium (55.3 × 12.7 × 12.7 mm) that supports 20 kgf. Other characteristics include: rated output = 1 ± 0.15 mV/V for nominal capacity (20 kgf), non-linearity = 0.05% FS (Full Scale), hysteresis = 0.05% FS, output impedance = 1000 Ω, overload = 150% nominal capacity. The use of bending beam load cells seems awkward when compared to inline/axial load cells but the truth is that they are much cheaper making them the best choice for low-cost systems.

**Figure 4:**
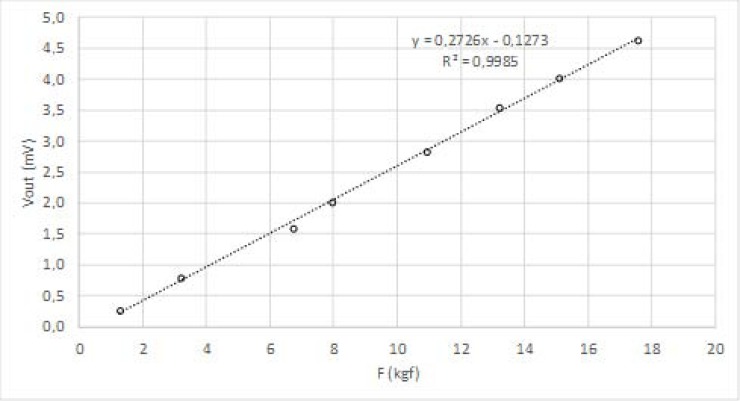
Load cell characterization.

The load cells were characterized using the fastening scheme shown in Figure 3b. Three legs of the walker were laid on the floor while the fourth leg was laid on a precision scale, model Spider 1S–60S from Mettler Toledo, all properly levelled. Static loads were applied to the walker frame by putting lead weights on it. The weight measured by the scale is equal to the force applied on the walker leg provided the leg does not move. The load cell under test was supplied with 5 V its output voltage was read using a 6½ digits digital multimeter, model 2701 from Keithley. Figure 4 presents the results obtained for a given load cell (similar results were obtained for the other cells). 

Using linear interpolation, validated by an R-squared value close to 1, the following relationship was found between the output voltage in mV (*Vout*) and the input force in kgf (*F*):

(6) Vout=0.2726×F−0.1273

The offset error is strongly attenuated by the subtractive nature of Equations (1) and (2). The remaining error is cancelled by running a self-calibration routine (see Section 3.5.1 for more details).

### 3.2. LIDAR

A low-cost LIDAR device [28] was used to perform distance measurements. The LASER associated with this device makes use of pulsed light and modulation techniques to improve the measurement accuracy. The main characteristics of the LIDAR include: working wavelength of 905 nm, maximum pulse train length of 256 pulses, pulse repetition rate of 20 kHz, accuracy better than 2.5 cm, measurement range up to 40 m (much higher than the required for our application), I2C communication interface, PWM output signal with 3 V of amplitude and 10 µs of resolution.

**Figure 5:**
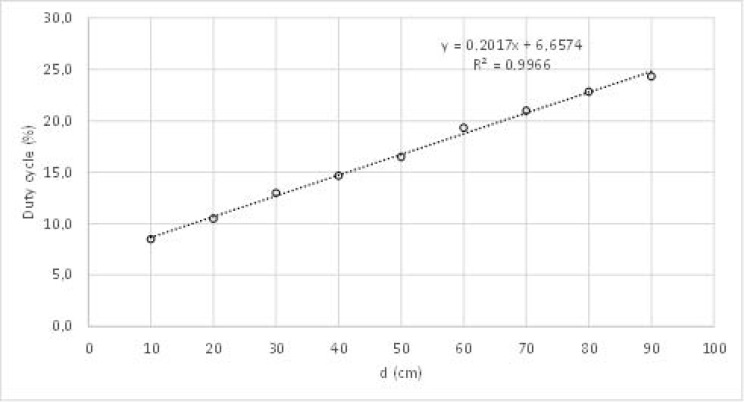
LIDAR characterization.

The characterization of the LIDAR was performed pointing the LASER to a moving target and measuring the duty cycle (δ) of the PWM signal. The sensor was fixed at one end of a graduated ruler while the target could move freely anywhere from 10 to 90 cm. The distance was measured directly from the ruler, with a resolution of 0.5 mm and the pulse width was measured using a universal counter, model PM6669 from Philips (Amsterdam, Netherlands). Figure 5 presents the obtained results.

Using linear interpolation, validated by an R-squared value close to 1, the following relationship was found between the duty cycle in % (*δ*) and the distance in cm (*d*):

(7) δ=0.2017×d+6.6574

### 3.3. Signal Conditioning

Each load cell is supplied at 5 V and conditioned by an instrumentation amplifier with gain 100 (AD623, Analog Devices, Norwood, MA, USA), followed by a non-inverting amplifier with gain 8 (MCP6272, Microchip, Chandler, AZ, USA) and an RC low-pass filter with a cut-off frequency of 20 Hz. The result is a smooth output swing from 0 to 4 V for forces between 0 and 20 kgf.

The PWM signal of the LIDAR passes through a similar filter (RC, low-pass, 20 Hz) and a non-inverting amplifier with gain 5, giving an output swing from 1 to 4 V for distances between 0 and 100 cm.

### 3.4. Data Acquisition

**Figure 6:**
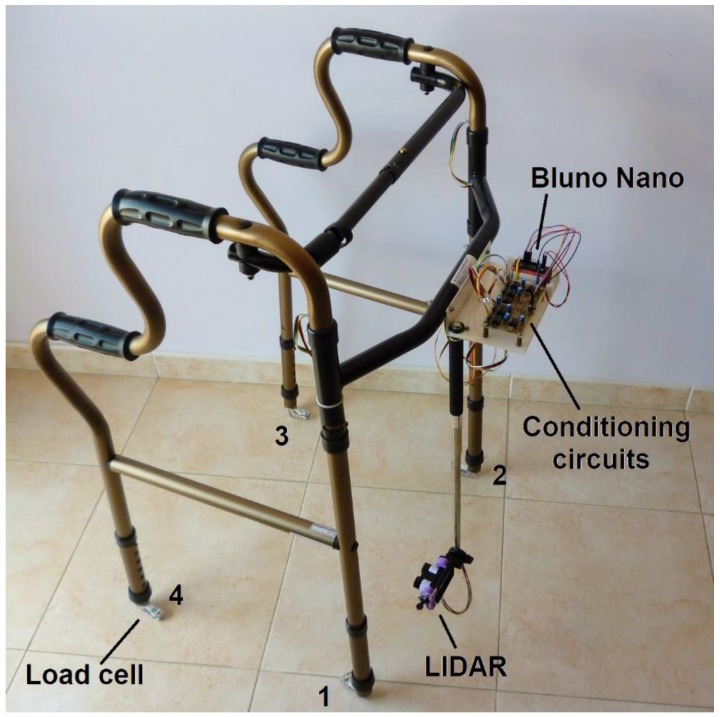
Instrumented walker.

Data is acquired by a Bluno Nano board [29] attached to the walker body as shown in Figure 6. The board provides all the resources of an Arduino Nano, plus a Bluetooth 4.0 wireless interface, all contained in a low-cost, small-footprint package.

The load cells are mounted on the walker legs, one on each leg and the LIDAR is suspended on a selfie-stick pointing to the injured foot. The five sensors (four load cells and the LIDAR) are sampled at 50 S/s and the resulting data stream is transmitted over the air to a host (computer or tablet) where it is processed. The Bluetooth link emulates a serial port on both sides allowing transmission speeds up to 1 Mbps.

It should be underlined the low cost of the proposed solution: the load cells (4 × 8 €), the LIDAR (150 €), the Bluno Nano board (40 €), the conditioning circuits (30 €) and some mechanical adapters (40 €) made a total of 292 €, a very reasonable price when compared to other proposals [30].

### 3.5. Data Processing

The samples arriving to the host are handled by a high-priority worker thread that buffers them into memory. Later, when on the main thread wakes up, every 125 ms in the present case, the samples are processed in batch, one by one. The buffer contains a variable number of samples, from 6 to a few more, depending on the host’s load at that moment. The state machine (responsible for classifying steps) is executed for all samples but the screen is refreshed only 8 times per second, which is enough to give the application good responsiveness and fluidity.

Data processing consists in the calculation of the risk indicators according to the following procedures:

1. Computation of *COF_x_* and *COF_y_* by solving Equations (1) and (2).
2. Computation of the first risk indicator (*I*_1_) by solving Equations (3) and (4).
3. Detection of “bad” steps (*B*) by running the state machine represented in Figure 2.
4. Computation of the second risk indicator (*I*_2_) by solving Equation (5).

**Figure 7:**
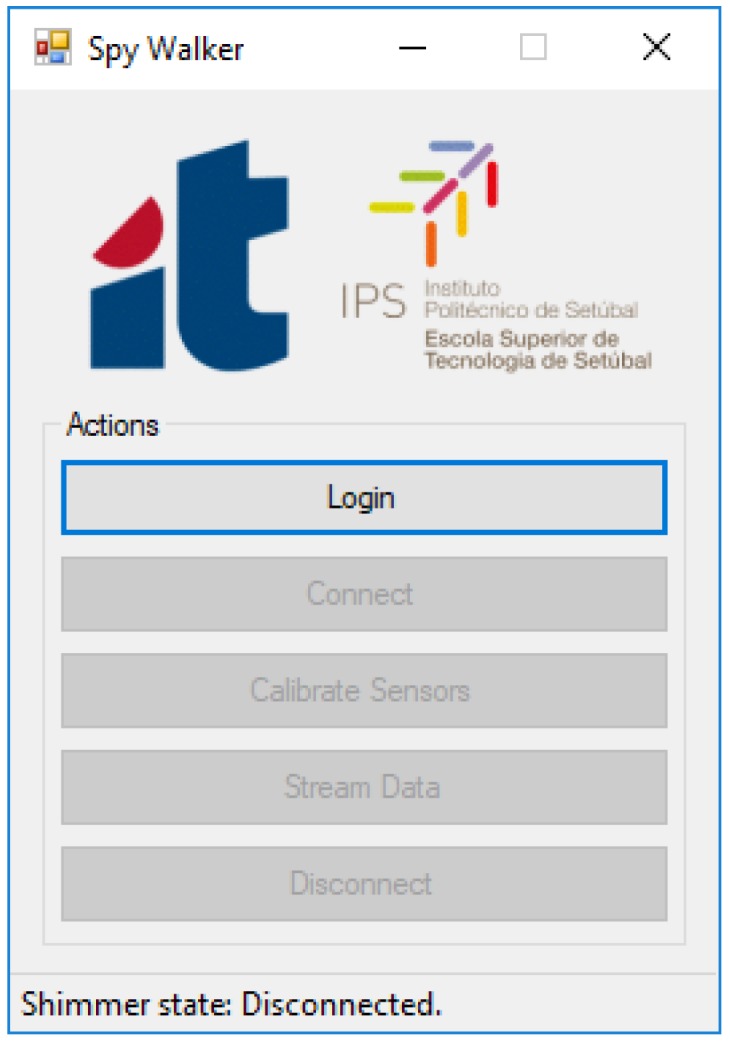
Main window of the Spy Walker application.

All these tasks are done by the application “Spy Walker” that runs on the host side and is managed by the physiotherapist. The application was developed in Visual Studio 2012 using the C# programming language. Its main window offers the following options (see Figure 7):

- Login: Ask for information about the user including its weight (needed to compute Equation (4)).
- Connect: Establish a Bluetooth link with the smart walker.
- Calibrate Sensors: Determine experimentally the offset error of the load cells and the walker weight (also needed for Equation (4)).
- Stream Data: Open a sub-window where the physiotherapist can monitor online the usage of the walker.
- Disconnect: Close the Bluetooth link with the smart walker.

#### 3.5.1. Calibrate Sensors

**Figure 8:**
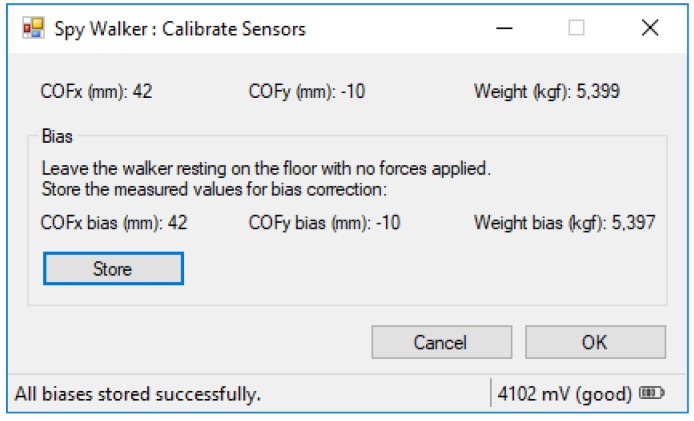
Calibrate Sensors sub-window.

The “Calibrate Sensors” sub-window (see Figure 8) serves to extract the offset error of the measurement system. With the walker resting on the floor, with no external forces applied, the user registers the *COF* coordinates and the weight of the walker frame. These residues are offset errors that will be cancelled later during normal operation. *COF* residues are subtracted from Equations (1) and (2), while the weight residue is subtracted from the numerator of Equation (4).

#### 3.5.2. Stream Data

**Figure 9:**
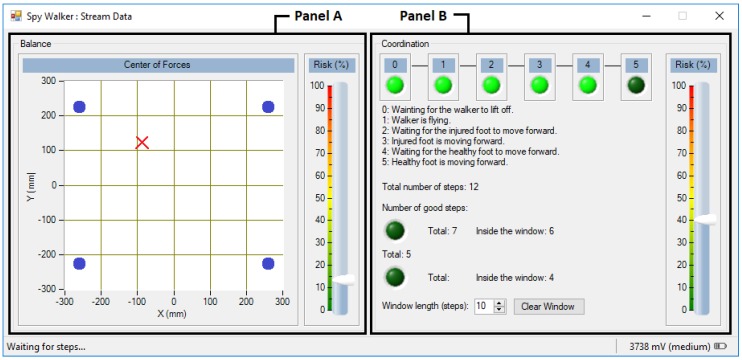
Stream Data sub-window. Panel A presents data related with force balance (the dark circles represent the walker legs) and panel B presents data related with motor coordination.

The “Stream Data” sub-window is where data processing takes place. The graphical interface is divided in two main panels as shown in Figure 9:

- Balance (panel A): The *COF* is computed and the result is presented as a cross moving over a *XY* graph. When the user loads his left side the cross moves toward negative values of *X*; when he loads his right side the cross moves toward positive values of *X*. The same applies for the front/back direction over the *Y* axis, much like a joystick. A vertical slider shows the instantaneous value of the *I*_1_ indicator.
- Coordination (panel B): The state machine is executed and the current gait phase is identified. A set of six LEDs are turned on sequentially as the state machine moves forward the next phase. If the user passes through all the phases successfully, all the LEDs end up lighted and the step is marked as “good”. If the user violates any phase, the machine is reset to the first stage (GP0) and the step is marked as “bad”. The number of “good” and “bad” steps is registered. A vertical slider shows the instantaneous value of the *I*_2_ indicator.

## 4. Experimental Results

This section describes the preliminary tests executed to verify the effectiveness of the measurement system and to get a first impression about the values of the risk indicators. A total of five tests were done involving signals from all sensors (load cells and LIDAR). The sensors were easily integrated in the commercial walker without need of any modification in its mechanical structure. The load cells were installed in the terminal part of the walker legs and the LIDAR was fixed in the frontal frame bar with the beam pointing to the user’s injured limb.

### 4.1. COF Location

**Figure 10:**
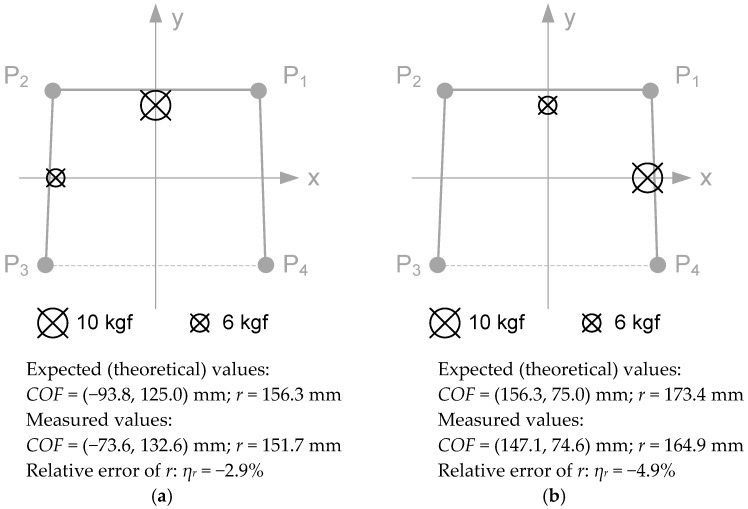
*COF* measurements after loading the walker frame with two lead weights: (**a**) 10 kgf at (0, 200) mm and 6 kgf at (−250, 0) mm; (**b**) 10 kgf at (250, 0) mm and 6 kgf at (0, 200) mm.

Two well-known lead weights were applied in precise locations of the walker frame to compare the *COF* measurements against the expected values. Figure 10 shows the two arrangements made: on the left, the 10 kgf weight was applied at the point (0, 200) mm and the 6 kgf weight was applied at the point (−250, 0) mm; on the right, the 10 kgf weight was applied at the point (250, 0) mm and the 6 kgf weight was applied at the point (0, 200) mm. For each arrangement, the measured value of *COF* and its distance to the origin (*r*) are presented, as well as the corresponding expected (theoretical) values. 

The relative error of r was chosen to assess the accuracy of the measurement system. The values obtained, below 5%, are acceptable taking into account that this is a lost-cost measurement system.

### 4.2. Computation of I_1_ (Assessment of Unbalance)

This test was done with the collaboration of a male user with 75 kgf of weight and impaired gait caused by an injury on the right lower limb. This user will be referred as “Wally” (code name) in the remaining of this paper.

**Figure 11:**
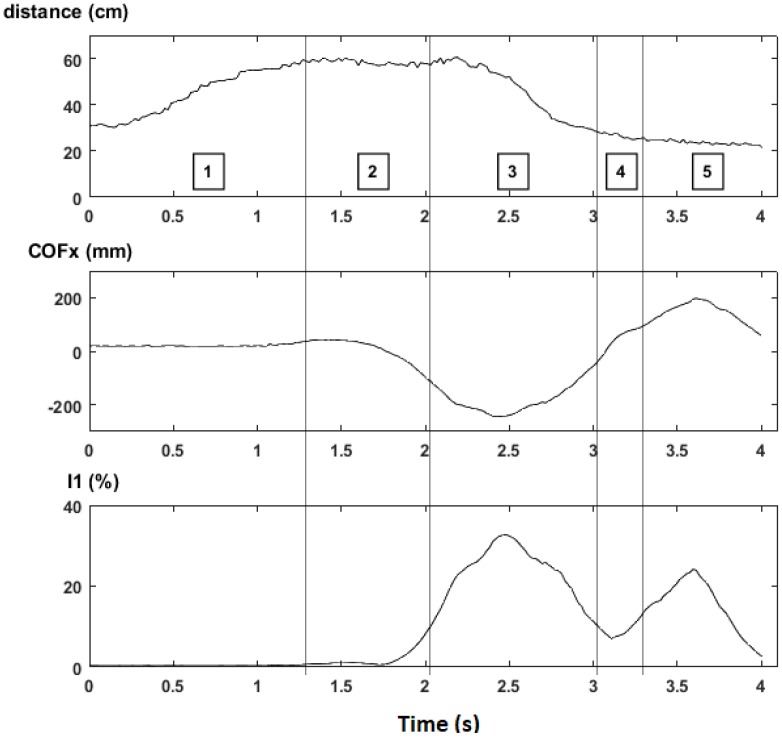
Synchronized plots of *d* (**top**), *COF_x_* (**middle**) and *I*_1_ (**bottom**) during a “good” step. The square boxes indicate gait phases from 1 to 5. The threshold values are WEIGHT_TH = 0.5 × WALKER_WEIGHT, TRAVEL_TH = 6 cm, LEFT_TH = −78 mm and RIGHT_TH = +78 mm.

Wally was instructed to make a “good” step following the recommendations of its physiotherapist. Figure 11 presents the resulting plots of *d*, *COF_x_* and *I*_1_, which can be explained as follows:

- Gait phase 0 was omitted because it is a waiting state.
- During gait phase 1 the walker is lifted in the air and travels about 30 cm (from *d* ≈ 30 cm to *d* ≈ 60 cm).
- Gait phase 2 is a waiting state that ends when the user starts moving the injured foot (making *COF_x_* cross LEFT_TH).
- During gait phase 3 the user transfers part of his weight to the left to compensate the lack of support on the injured foot [31]. While the injured foot moves forward, the distance returns to the base value (around 30 cm) and *COF_x_* returns to the origin, thus making the machine enter in stage 4.
- Gait phase 4 is another waiting state that ends when the user moves the healthy foot (making *COF_x_* cross RIGHT_TH).
- During gait phase 5 the user transfers part of his weight to the right. The load applied during phase 5 is lower than that applied on phase 3 because the healthy foot moves more easily than the injured foot. The step ends when both feet stay side-by-side, making *COF_x_* return to the origin and restart the state machine.

The risk indicator *I*_1_ has two maximums that match the extreme values of *COF_x_*, when the user is more unbalanced and relies more on the walker. The first maximum is above 30% and the second maximum is above 20%, meaning that the unbalance is greater when the user moves the injured foot. These results suggest that *I*_1_ is strongly related with the posture of the user inside the walker frame but more experiments, involving more impaired users, will be needed to identify patterns and establish threshold values for *I*_1_.

### 4.3. Computation of I_2_ (Assessment of Motor Incoordination)

This test was done with the collaboration of ten non-disabled, inexperienced users with ages between 42 and 70 years old. Since none of them have ever used a walker before, they all received a preliminary briefing, given by a physiotherapist, about how to use the device correctly (i.e., they all became aware of the gait phases depicted in Figure 2).

**Figure 12:**
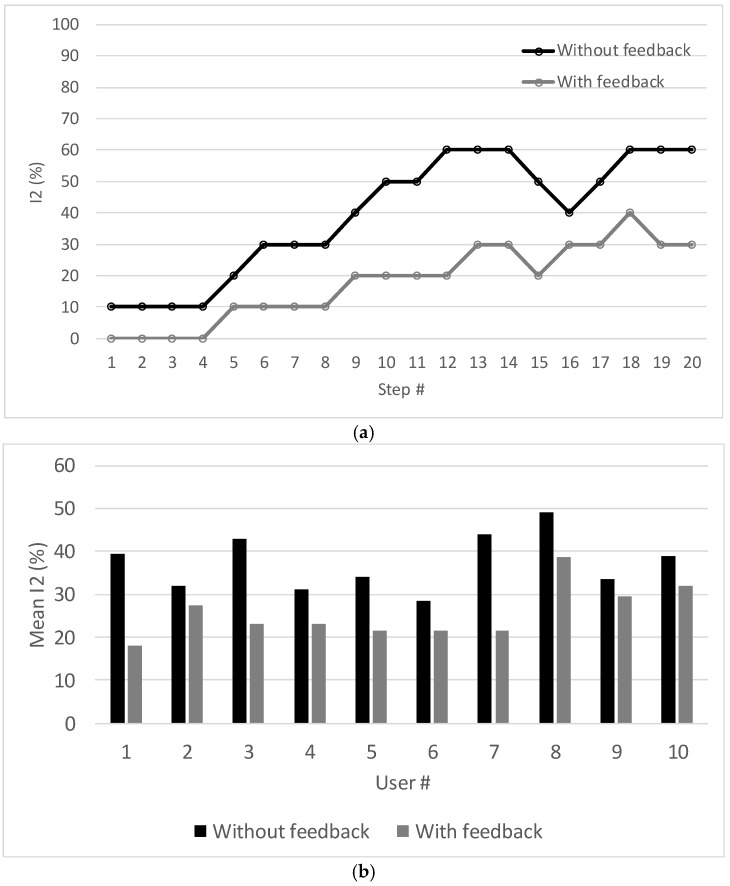
Risk indicator *I*_2_ with and without feedback from the Spy Walker application: (**a**) Instantaneous values for the user 1; (**b**) Mean values for all users.

Each user was instructed to do two walks, of twenty steps each, over a straight line drawn on the floor. During the first walk the user did not receive any feedback at all; during the second walk the user received live feedback (audio) from the Spy Walker application. The feedback included the classification of the step (“good” or “bad”) and the cause of the failure (“step aborted,” “injured foot failed to move forward” or “healthy foot failed to move forward”). Figure 12a plots the risk indicator *I*_2_ for user 1 during the two walks. Similar results were found for the other users. Figure 12b presents the average value of *I*_2_, per walk, with and without feedback, for the ten users. In all cases, the motor coordination became better after the feedback given by the Spy Walker application. 

The state machine worked well with no false “goods” and a few false “bads”. This can be justified by the fact that the validation of a “good” step is very demanding. Failures such as aborting a step by lifting the walker in the air or starting a step with the wrong foot were successfully detected.

### 4.4. COF Trajectories

**Figure 13:**
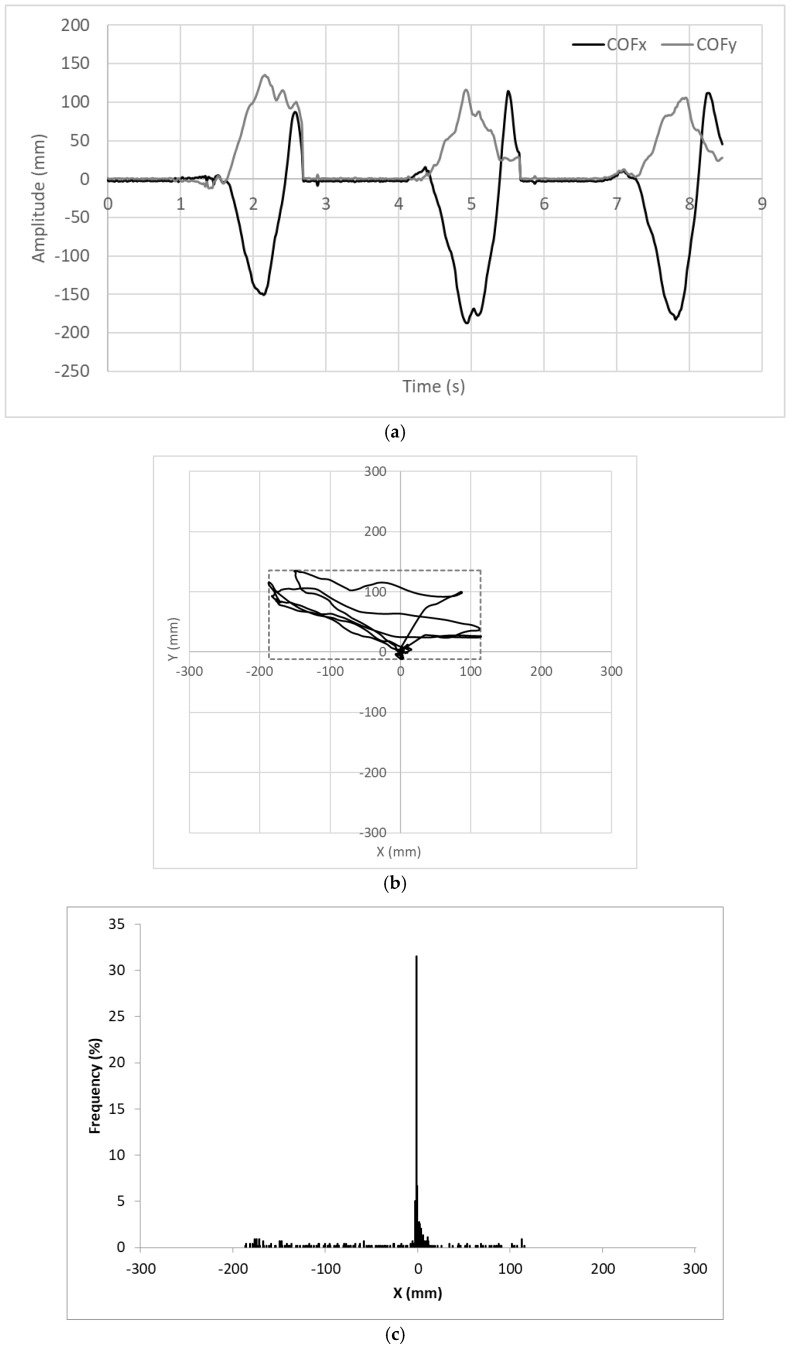
Behaviour of *COF* during a sequence of three “good” steps: (**a**) *COF_x_* and *COF_y_* over time; (**b**) trajectory of *COF* over the plane; (**c**) histogram of *COF_x_*.

Wally was instructed to make a sequence of three “good” steps. During that action, the coordinates of the *COF* were recorded and plotted as shown in Figure 13. The plot on the top (a) represents the waveforms of *COF_x_* and *COF_y_* over time; the plot in the middle (b) represents the trajectory of *COF* on the plane; and the plot at the bottom (c) represents the histogram of *COF_x_*. When the user is not walking, which is most of the time, the *COF* is around the origin. When the user makes a step, the *COF* moves first to the 2nd quadrant, then to the 1st quadrant and finally returns to the origin. 

The centre of the rectangle depicted in Figure 13b is located in (−37, 62) mm because the area covered on the 2nd quadrant is wider than that covered on the 1st quadrant. The skewness of the statistical distribution shown in Figure 13c is negative, equal to −0.93, because the tail on the left side is longer than that on the right side. These are mathematical parameters that suggest (correctly) that the user has an injury on the right lower limb and, for that reason, needs more support on his left side.

### 4.5. LIDAR Measurements

**Figure 14:**
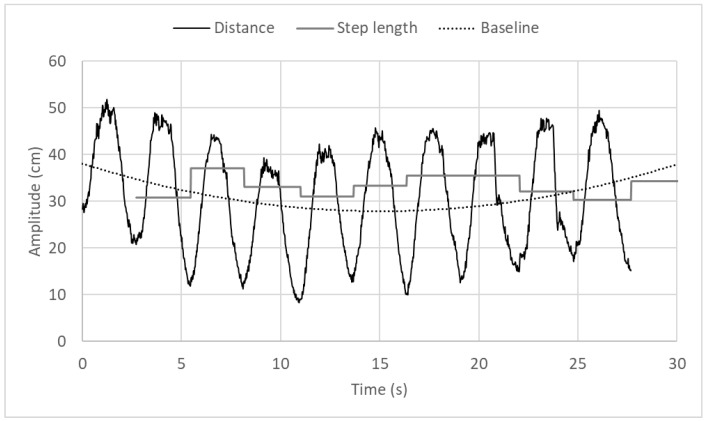
Distance values and step lengths for walker gait containing 10 steps.

Wally was instructed to make a sequence of ten “good” steps. During that action, the distance measured by the LIDAR, together with the corresponding step lengths, were recorded and plotted as shown in Figure 14. The step length is calculated by subtracting the maximum distance value (acquired at the end of gait phase 1) from the minimum distance value (acquired at the end of phase 5). 

The user walked a total distance of 3.33 m, in steps varying between 37.1 cm and 30.3 cm, with mean value equal to 33.3 cm and standard deviation equal to 2.3 cm. The low value of standard deviation suggests a regular walking pattern. The baseline of the LIDAR signal indicates the average distance between the user and the walker frame.

## 5. Conclusions

The paper presents an upgraded solution that combines force and distance measurements to monitor the usage of walker assistive devices. The sensed data is transmitted over a Bluetooth link and processed on the host side to extract two risk indicators: *I*_1_ representing the (un)balance of forces applied on the walker legs and *I*_2_ representing the (in)coordination between walker movements and user gait. Data processing and presentation is done by a Windows-based application managed by the physiotherapist.

The experimental results showed that the measurement system works as expected concerning the localization of the *COF* and the extraction of distance metrics. The risk indicators were computed successfully and the obtained values seem to be meaningful to assess the posture of the user inside the walker frame. The live feedback provided by the Spy Walker application can help inexperienced users to manoeuvre correctly walker assistive devices. New mathematical indicators (geometrical and statistical) may be extracted to characterize gait and infer about the physical condition of the user.

It is important to refer that the proposed measurement system is a low-cost prototype that can easily be adapted to existing mobility aiding devices, including standard walkers, wheeled walkers and rollators.
